# Variation in the calorific values of different plants organs in China

**DOI:** 10.1371/journal.pone.0199762

**Published:** 2018-06-28

**Authors:** Pu Yan, Li Xu, Nianpeng He

**Affiliations:** 1 Key Laboratory of Ecosystem Network Observation and Modeling, Institute of Geographic Sciences and Natural Resources Research, Chinese Academy of Sciences, Beijing, China; 2 College of Resources and Environment, University of Chinese Academy of Sciences, Beijing, China; Chinese Academy of Forestry, CHINA

## Abstract

The calorific value (CAL, KJ g^–1^) of different plant organs are important plant traits. Variation in CAL among different plant organs reflects the energy utilization and distribution strategy of plant. Here, we explored how the CAL of different plant organs varies at the species and regional level in relation to vegetation type (3697 forests samples, 430 grasslands samples, and 146 deserts samples). The results showed that, in the forests and grasslands, CAL significantly differed among the different organs and functional types of plants. The CAL of different organs in forests was ordered as: leaf (19.71 ± 1.82 KJ g^–1^) > branch (19.33 ± 1.32 KJ g^–1^) > trunk (19.09 ± 1.44 KJ g^–1^) > root (19.02 ± 1.11 KJ g^–1^). For forests, the CAL of plant organs, except for the leaves of trees and shrubs, increased with increasing latitude (*P* < 0.01). In comparison, the CAL in the roots of shrubs and herbs decreased with increasing longitude (*P* < 0.01). Through delineating systematic references of CAL among different plant organs, our findings provide key parameters to improve estimates of biomass energy at regional and global scales.

## Introduction

The calorific value (CAL, KJ g^–1^) of plant organs is the quantity of energy that is released by the complete combustion of dry matter per unit mass. CAL is an important plant trait because it reflects the photosynthetic ability (gross primary productivity) and nutritional status of plants to some extent [1]. Moreover, changes to CAL among different plant organs might reflect differences in energy storage strategies. Therefore, understanding how CAL varies among different plant organs and across regions could provide insights on the energy utilization and distribution strategy of plants.

Some studies have explored the characteristics of CAL in different plant organs at local scales. For instance, Long et al. [2] revealed the distribution of CAL in sunflower leaves in comparison to that in other organs. Song et al. [3] analyzed the spatial pattern of CAL in the leaves of trees across China and the factors that influence this pattern. However, most studies tend to focus on specific plants or specific organs (e.g., leaf, root) [2–11]. Because of the inconsistency and paucity of data on CAL, few studies have explored how CAL varies among different plant organs or among different vegetation types, especially at large scales.

Different plant organs (leaf, stem, trunk, and root) have different morphological characteristics and functional traits [12–19]. For example, we know that the difference between needles and broadleaves is one of the most important examples of plant phenotypic dissimilarity. In addition, it is generally assumed that the leaf organ is mainly responsible for photosynthesis, while the root system is mainly responsible for the absorption of inorganic salts and moisture. This division of labor is a strategy adopted by plants over long-term evolution to adapt to the complex and changing environment. Yet, maybe plants utilize different strategies to distribute energy across different organs to help plant optimize energy use. For instance, Song et al. [20] showed that CAL and the element content (especially the carbon content) are more strongly correlate some plants, with different functional traits often leads to noticeable differences in element content. Therefore, we hypothesized that the CAL of different plant organs significantly differs across different vegetation types.

**Fig 1:**
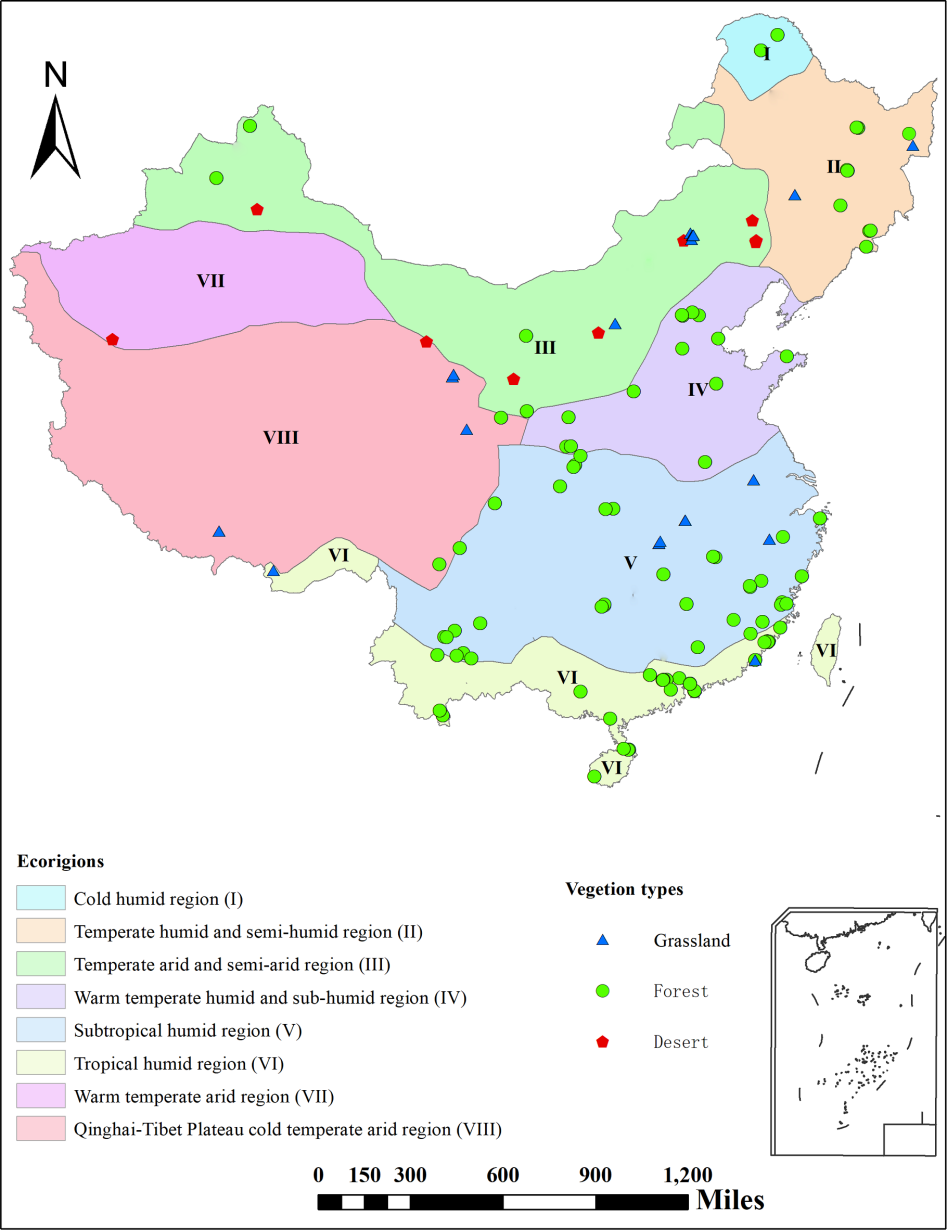
Spatial distribution of sampling sites for the collection of plant caloric values (CAL, KJ g^–1^) in different vegetation types in China.

To explore how CAL varies among different plant organs and different vegetation types at a large scale, we collected samples from vegetation in Chinese terrestrial ecosystems (forests, grasslands, wetlands, and deserts) to assimilate a CAL dataset from field sampling and literature collection (Fig 1). The main objectives of this study were to: 1) determine how CAL differs among different plant organs and vegetation types; 2) explore regional differences in the CAL of plant organs and how it is distributed in plants along with the factors that influence the distribution of CAL. Our results are expected to provide a systematic reference of plant CAL to improve regional estimates of biomass energy in the future.

## Data and methods

### Data sources

#### Data on CAL

Chinese terrestrial ecosystem is the research area in this study. Data on CAL were collected through two approaches: the experimental measurement data by our team and data from the published literature. For the experimental data, we sampled the leaves of 1124 plant species that were healthy, fully expanded, and sun-exposed. The plant samples were collected along the North-South Transect of Eastern China (NSTEC) in July and August 2013 [21]. We measured leaf CAL using a Parr 6300 automatic isoperibol calorimeter (Parr Instrument Company, Moline, IL, USA). We collected data from the published literature using the Institute for Scientific Information (ISI) (http://apps.webofknowledge.com) and China National Knowledge Internet (http://www.cnki.net) databases, using “plant” and “calorific value” as key words to search for articles. The articles were further screened based on the following criteria: 1) data on CAL should be obtained through field measurements rather than model simulations; and 2) basic information (e.g., sampling location, time, vegetation types, life forms, and CAL of various plant organs [leaf, trunk, branch, bark, and root]) should be clearly presented. Then, the two datasets were combined and the units were transformed to KJ g^–1^. To enhance the credibility of data and to reduce uncertainty, we excluded all samples with CAL exceeding a mean ± 3 times the standard deviation to remove the adverse impacts of outliers during data analysis [22]. After data integration, data for this study included 3697 forest samples, 430 grassland samples, and 146 desert samples (Fig 1 and S1 Table; S1 and S2 Figs).

#### Data on ecological regions and climate

Based on climate and topography [23], the terrestrial ecosystems of China were divided into eight regions: cold humid region (I), temperate humid and semi-humid region (II), temperate arid and semi-arid region (III), warm temperate humid and sub-humid region (IV), subtropical humid region (V), tropical humid region (VI), warm temperate arid region (VII), and Qinghai-Tibet Plateau cold temperate arid region (VIII). Climate data (mean annual temperature [MAT, °C] and mean annual precipitation [MAP, mm]) were obtained from the National Data Sharing Infrastructure of Earth System Science (http://www.geodata.cn/). We used the tool “Extract Multi Values to points” in ArcGIS software to extract the corresponding MAT and MAP values for each site.

### Calculation

Data on plant CAL were analyzed at the species and regional level. At the species level, we summarized all plants genotypes across China based on "Chinese Flora" [24]. First, vegetation types were divided into forests, grasslands, and deserts. Then, plants in forests were separated into three plant functional groups (PFGs; trees, shrubs, and herbs). Plants in grasslands and deserts were mainly herbs. To explore how CAL varies among different plant life forms, forest plants were divided into needle trees and broadleaved trees, and evergreen trees and deciduous trees. The organs of trees were divided into four components: leaves, branches, trunks, and roots. For shrubs, plants were divided into leaves, branches, and roots. For herbs, plants were divided into leaves, stems, and roots. In grasslands, plants were divided into aboveground and underground parts. Eq 1 was used to calculate the CAL of different organs among the different vegetation types after the data were compiled.

At the regional scale, we used the arithmetic average to calculate the CAL (KJ g^–1^) of various organs of different vegetation types across eight ecological regions (Eq 2). To reduce uncertainty caused by sample quantity, we used the national average for ecological regions when the sample number was less than 10.

$$\text{CAL}\_\text{organ}=\sum_{\text{i}=1}^{\text{n}}{\text{CAL}}_{\text{i}}/\text{n}$$ (1)

$$\text{CAL}\_\text{region}=\sum_{\text{j}=1}^{\text{k}}{\text{CAL}}_{\text{j}}/\text{k}$$ (2)

where, CAL_–organ_ represents the national average of organ at species level, CAL_i_ represents the CAL (KJ g^–1^) of each sample, i represents organs (leaf, branch, trunk, and root), n represents the number of organ samples; CAL_–region_ is the CAL of plant organs at the regional level, CAL_j_ represents the CAL of each sample in the region, and k represents the CAL of plant organs in the region.

### Statistical analysis

One-way analysis of variance (ANOVA) was used to analyze differences in CAL among various organs and regions. Least multiple comparison (LSD) was used to explore the difference in CAL among trees, shrubs, and herbs. The independent-samples T test was used to test the differences in CAL among the different plant life forms of trees. The latitudinal and longitudinal patterns of plant CAL for different organs were analyzed using the linear regression of the ordinary least squares method (OLS). Pearson correlation analysis was used to analyze the correlation between plant CAL at the organ level and climatic factors (MAT and MAP). The significant of the statistical tests was set as *P* = 0.05. All data were analyzed with ArcGIS (Version 10.2, ESRI, USA), SPSS 18.0 (SPSS Inc., Chicago, IL, USA, 2004) and Sigmaplot 12.5 (Washington, IL, USA, 2006) software.

## Results

### Differences in CAL among different plant organs

#### Forests vegetation

**Fig 2:**
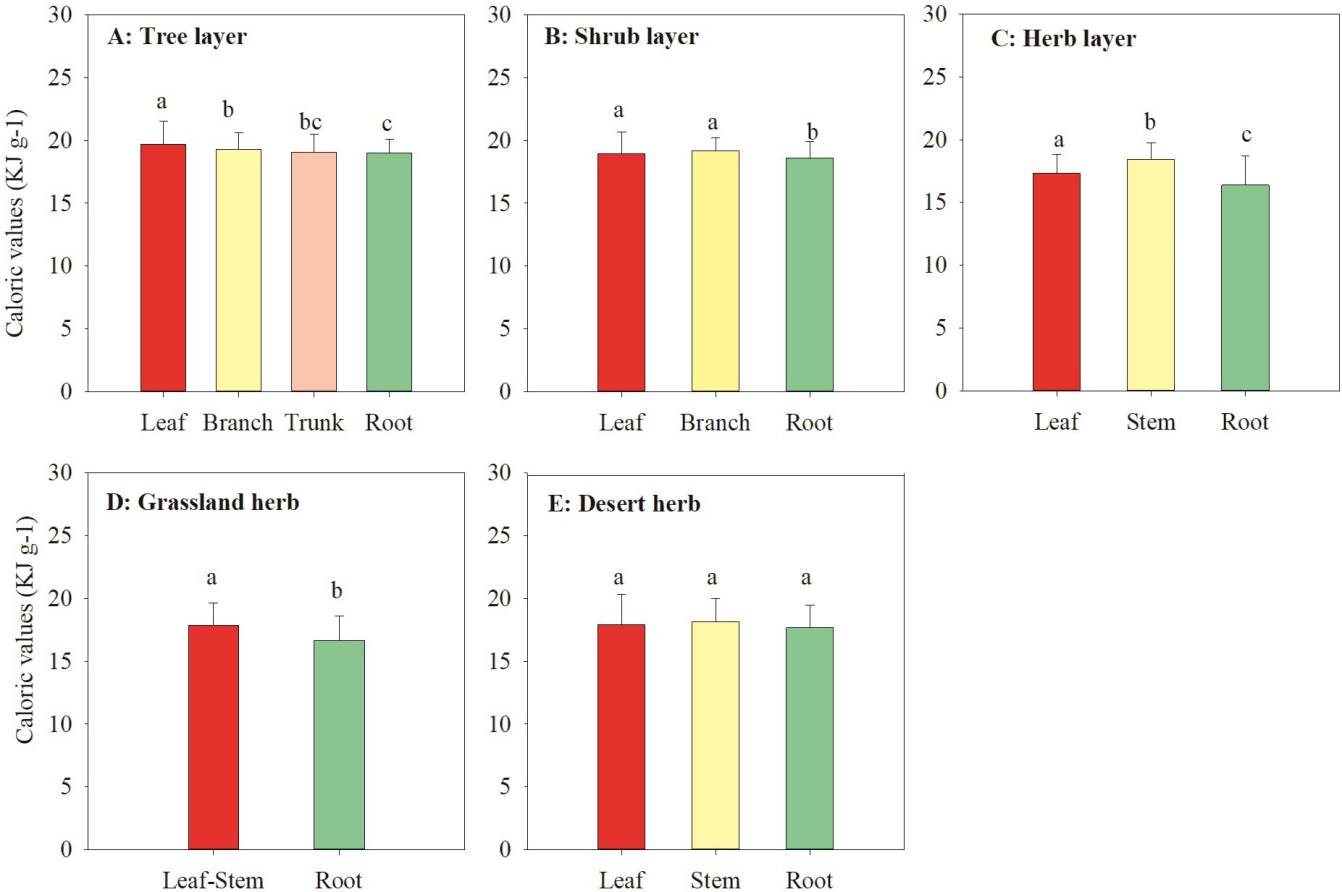
Caloric values (CAL, KJ g^–1^) of different plant organs among the different vegetation types. Different letters indicate significant differences at the *P* = 0.05 level.

**Fig 3:**
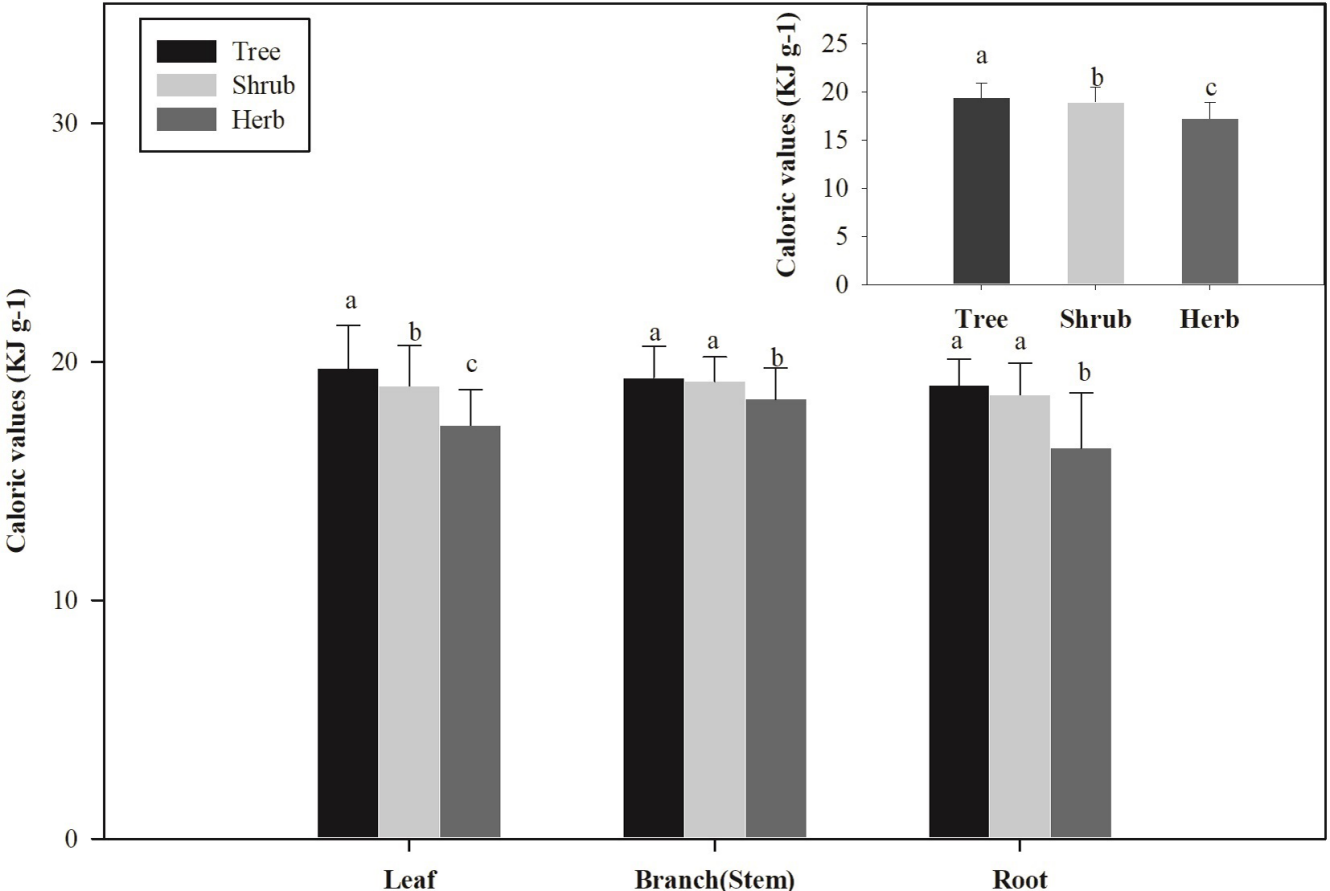
Changes in the caloric values (CAL, KJ g^–1^) among the three plant functional groups. Different letters indicate significant differences at the *P* = 0.05 level.

There were significant differences in the CAL of different plant organs in forests (*P* < 0.05; Fig 2A–2C). In addition, there was a significant difference in CAL among different organs and different PFGs (trees, shrubs, and herbs) (*P* < 0.05; Fig 3).

**Fig 4:**
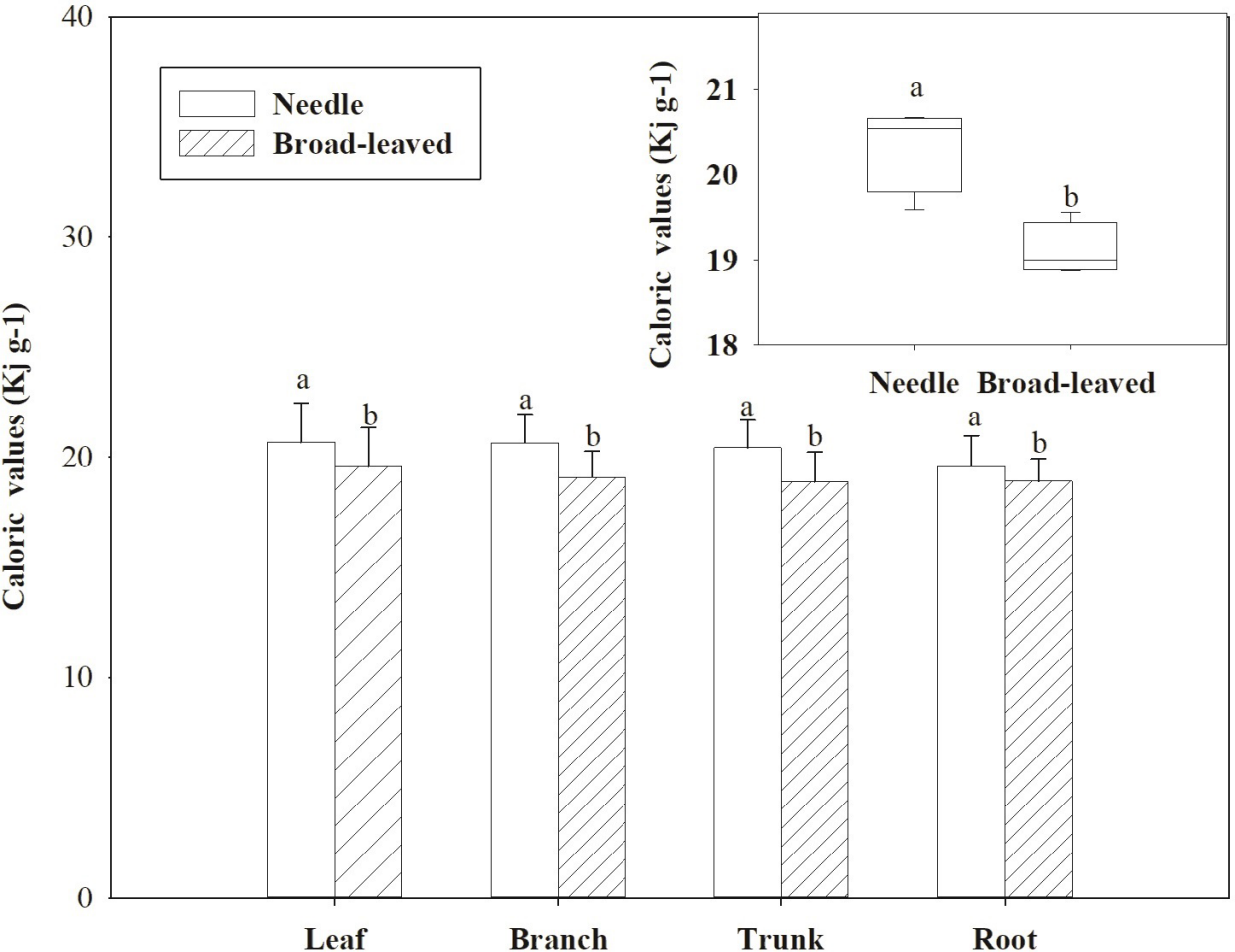
Changes in the caloric values (CAL, KJ g^–1^) of various plant organs in the two plant life forms. Different letters indicate significant differences in each cluster at the *P* = 0.05 level.

For trees, there were significant differences in CAL among various organs (leaf, branch, and root) (*P* < 0.05; Figs 2A and S3). CAL was ordered: leaf (19.71 ± 1.82 KJ g^–1^) > branch (19.33 ± 1.32 KJ g^–1^) > trunk (19.09 ± 1.44 KJ g^–1^) > root (19.02 ± 1.11 KJ g^–1^). Furthermore, the CAL of needle trees were higher than that of broadleaf trees (*P* < 0.05; Fig 4), but there was no significant difference between evergreen and deciduous trees (*P* > 0.05; S4 Fig). Similar to the tree layer, the CAL in aboveground parts (leaf and stem) of the shrub and herb layer was significantly higher than that of the underground parts (root) (*P* < 0.05; Fig 2B and 2C). Overall, the CAL of different PFGs was ordered: tree > shrub > herb (*P* < 0.05; Fig 3).

#### Grasslands and deserts vegetation

For grassland vegetation, the CAL of the aboveground biomass (17.89 ± 1.77 KJ g^–1^) was significantly higher than that of the underground biomass (16.68 ± 1.95 KJ g^–1^) (*P* < 0.05; Fig 2D). However, for desert vegetation, there was no significant difference in CAL among various organs (Fig 2E).

### Changes to plant CAL among regions

The results showed that there were no significant differences in CAL for most of plant organs in forest across various regions (*P* > 0.05; Table 1). However, the CAL of the branch and trunk in the tree layer was significantly different (*P* < 0.05; Table 1), except for the temperate arid and semi-arid region (III), subtropical humid region (V), and Qinghai-Tibet Plateau cold temperate arid region (VIII). For grasslands and deserts, there was no significant difference in the CAL of plant organs in different regions (*P* > 0.05; Table 2), except for the aboveground CAL of grasslands.

**Table 1:** Caloric values (CAL, KJ g^–1^) of different plant organs in the forests of the eight ecological regions in China.

	Organs		Caloric values (CAL, KJ g^–1^)							
			I^§^	II	III	IV	V	VI	VII	VIII
Trees	Leaf	Mean	19.71^ns^^‡^	19.58^ns^	**19.71**^¶^	19.37^ns^	**19.71**	19.73^ns^	19.70^ns^	**19.71**
		SD^†^	1.03	1.98	**1.82**	1.92	**1.82**	1.74	1.90	**1.82**
		N	22	84	**931**	92	**931**	599	119	**931**
	Branch	Mean	20.07^ab^	19.92^ab^	**19.33**	18.96^a^	**19.33**	19.27^ab^	19.21^b^	**19.33**
		SD	1.32	1.34	**1.32**	1.81	**1.32**	1.25	0.73	**1.32**
		N	12	27	**494**	54	**494**	314	78	**494**
	Trunk	Mean	20.74^bc^	19.89^ac^	18.97^a^	19.04^ab^	**19.09**	18.88^a^	19.27^a^	**19.09**
		SD	1.35	1.47	0.86	1.66	**1.44**	1.52	0.80	**1.44**
		N	12	38	71	45	**552**	300	81	**552**
	Root	Mean	**19.02**	19.66^ns^	**19.02**	19.40^ns^	**19.02**	18.81^ns^	19.12^ns^	**19.02**
		SD	**1.11**	0.99	**1.11**	1.15	**1.11**	1.06	0.62	**1.11**
		N	**273**	21	**273**	19	**273**	183	46	**273**
Shrubs	Leaf	Mean	19.54^b^	19.13^ab^	**18.99**	18.59^ab^	**18.99**	18.95^ab^	19.23^ab^	19.96^b^
		SD	1.65	1.68	**1.71**	1.38	**1.71**	1.79	1.43	2.34
		N	22	50	**599**	77	**599**	343	87	14
	Branch	Mean	**19.18**	20.52^ns^	19.53^ns^	18.91^ns^	**19.18**	19.01^ns^	19.11^ns^	**19.18**
		SD	**1.03**	0.41	0.70	1.28	**1.03**	1.10	0.54	**1.03**
		N	**252**	12	36	32	**252**	117	50	**252**
	Root	Mean	**18.62**	**18.62**	**18.62**	19.59^ab^	**18.62**	18.11^a^	19.06^ab^	**18.62**
		SD	**1.32**	**1.32**	**1.32**	1.04	**1.32**	1.31	0.74	**1.32**
		N	**150**	**150**	**150**	11	**150**	87	40	**150**
Herbs	Leaf	Mean	17.89^ns^	17.57^ns^	16.83^ns^	17.22^ns^	**17.34**	17.29^ns^	**17.34**	**17.34**
		SD	1.08	1.26	1.15	1.49	**1.49**	1.68	**1.49**	**1.49**
		N	30	86	31	89	**381**	136	**381**	**381**
	Stem	Mean	**18.44**	**18.44**	**18.44**	**18.44**	**18.44**	18.87	**18.44**	**18.44**
		SD	**1.32**	**1.32**	**1.32**	**1.32**	**1.32**	0.82	**1.32**	**1.32**
		N	**25**	**25**	**25**	**25**	**25**	18	**25**	**25**
	Root	Mean	**16.39**	**16.39**	16.44^ns^	**16.39**	**16.39**	16.17^ns^	**16.39**	**16.39**
		SD	**2.30**	**2.30**	0.72	**2.30**	**2.30**	2.81	**2.30**	**2.30**
		N	**40**	**40**	10	**40**	**40**	22	**40**	**40**

^†^ SD, standard deviation; N, number of plant species;

^‡ ^the same letter “a” or “b” and “ns” represent no significant difference, different letters “a” and “b” represent significant differences at the *P* = 0.05 level;

^§^I, Cold humid region; II, Temperate humid and semi-humid region; III, Temperate arid and semi-arid region; IV, Warm temperate humid and sub-humid region; V, Subtropical humid region; VI, Tropical humid region; VII, Warm temperate arid region; VIII, Qinghai-Tibet Plateau cold temperate arid region.

^¶^The number of the bold font indicates that the national average is used instead.

**Table 2:** Caloric values (CAL, KJ g^–1^) of different plant organs in the grasslands and deserts of the eight ecological regions in China.

	Functional groups	Organs		Caloric values (CAL, KJ g^–1^)							
				I^§^	II	III	IV	V	VI	VII	VIII
Grasslands	Herb		Mean	**17.89**^¶^	18.19^a^^‡^	17.55^ab^	**17.89**	**17.89**	16.13^a^	**17.89**	18.83^b^
		Aboveground	SD^†^	**1.77**	1.43	1.39	**1.77**	**1.77**	1.55	**1.77**	2.00
			N	**342**	78	134	**342**	**342**	35	**342**	90
			Mean	**16.68**	16.91^ns^	16.31^ns^	**16.68**	**16.68**	**16.68**	**16.68**	**16.68**
		Underground	SD	**1.95**	2.18	0.86	**1.95**	**1.95**	**1.95**	**1.95**	**1.95**
			N	**88**	58	22	**88**	**88**	**88**	**88**	**88**
Deserts	Herb		Mean	**17.93**	**17.93**	**17.93**	18.03	**17.93**	**17.93**	**17.93**	**17.93**
		Leaf	SD	**2.40**	**2.40**	**2.40**	2.36	**2.40**	**2.40**	**2.40**	**2.40**
			N	**61**	**61**	**61**	59	**61**	**61**	**61**	**61**
			Mean	**18.17**	**18.17**	**18.17**	18.21	**18.17**	**18.17**	**18.17**	**18.17**
		Stem	SD	**1.80**	**1.80**	**1.80**	1.88	**1.80**	**1.80**	**1.80**	**1.80**
			N	**46**	**46**	**46**	42	**46**	**46**	**46**	**46**
			Mean	**17.68**	**17.68**	**17.68**	17.51	**17.68**	**17.68**	**17.68**	**17.68**
		Root	SD	**1.80**	**1.80**	**1.80**	1.77	**1.80**	**1.80**	**1.80**	**1.80**
			N	**39**	**39**	**39**	35	**39**	**39**	**39**	**39**

^†^ SD, standard deviation; N, number of samples;

^‡^ the same letter “a” or “b” and “ns” represent no significant difference, different letters “a” and “b” represent significant differences at the *P* = 0.05 level;

^§^I, Cold humid region; II, Temperate humid and semi-humid region; III, Temperate arid and semi-arid region; IV, Warm temperate humid and sub-humid region; V, Subtropical humid region; VI, Tropical humid region; VII, Warm temperate arid region; VIII, Qinghai-Tibet Plateau cold temperate arid region.

^¶^The number of the bold font indicates that the national average is used instead.

### Spatial patterns in plant CAL

#### Latitudinal patterns

**Fig 5:**
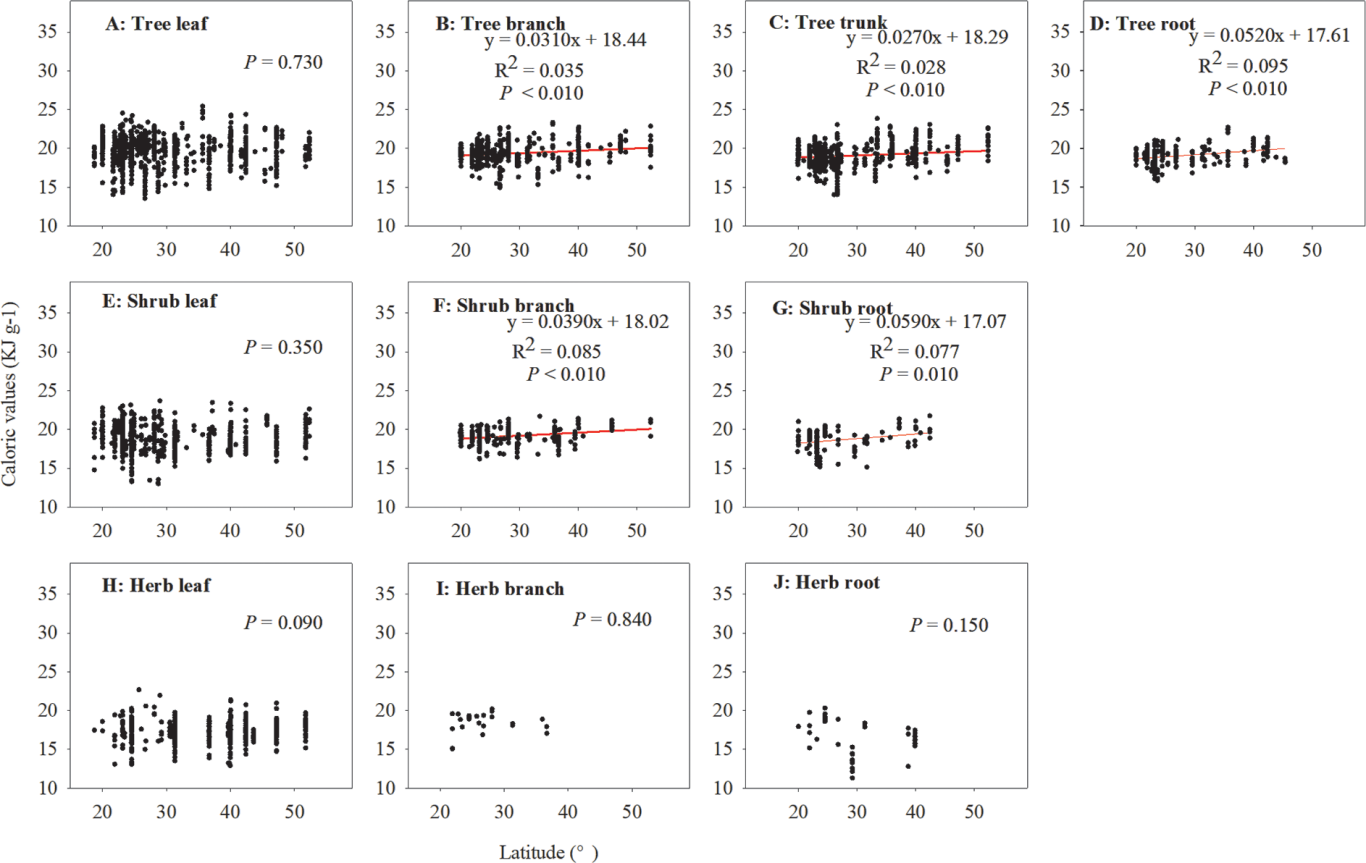
Latitudinal patterns of caloric values (CAL, KJ g^–1^) of different plant organs in the forests.

For forests, collectively, our results showed that, except for the leaves, the CAL of various organs in the tree and shrub layer increased with increasing latitude (*P* < 0.01; Fig 5). However, there was no apparent latitudinal pattern for leaf, branch, and root CAL in the herb layer. For deserts and grasslands, no significant latitudinal pattern in the CAL of different plant organs was detected.

#### Longitudinal patterns

Compared to latitudinal patterns, the CAL of leaves in the tree layer decreased with increasing longitude (*P* < 0.01; Panel A in S5 Fig). In the shrub layer, the CAL of branch increased with increasing longitude (*P* < 0.01), whereas the CAL of roots decreased with increasing longitude (*P* < 0.01; Panel F and G in S5 Fig). In the herb layer, the CAL of roots decreased with increasing longitude (*P* < 0.01; Panel J in S5 Fig).

For grasslands, the CAL of the aboveground biomass decreased with increasing longitude (*P* < 0.01), while the CAL of the underground biomass showed no longitudinal patterns (Panel D and E in S6 Fig). For deserts, no longitudinal pattern was detected for the CAL of organs (Panel A–C in S6 Fig).

### Influence of climatic factors on plant CAL

MAT had a significant effect on the CAL of plant organs in forests (*P* < 0.001); however, there was no significant correlation between MAT and the leaves of trees and herbs (Table 3). For grasslands and deserts, MAT had no significant effect on the CAL of plant organs (except for herb aboveground parts) (*P* > 0.05; Table 3).

**Table 3:** Regression relationships between caloric values (CAL, KJ g^–1^) of different plant organs and mean annual temperature (MAT) of different ecosystem types.

	Functional groups	Organs	Regression equations	R^2^	*P*
Forests	Tree	Leaf	–	–	0.789
		Branch	y = –0.0400x + 19.95	0.039	< 0.001
		Trunk	y = –0.0420x + 19.70	0.045	< 0.001
		Root	y = –0.0710x + 20.17	0.170	< 0.001
		Leaf	y = 0.0170x + 18.74	0.007	0.049
	Shrub	Branch	y = –0.0450x + 19.83	0.087	< 0.001
		Root	y = 0.0320x + 17.52	0.004	< 0.001
		Leaf	–	–	0.330
	Herb	Stem	–	–	0.616
		Root	–	–	0.903
Grasslands	Herb	Aboveground	y = –0.1130x + 18.23	0.130	< 0.001
		Underground	–	–	0.229
		Leaf	–	–	0.310
Deserts	Herb	Stem	–	–	0.387
		Root	–	–	0.695

For forests, CAL had a negative linear correlation with MAP for most organs in the tree and shrub layers (*P* < 0.01; Table 4). In contrast, for grasslands and deserts, the relationships between the CAL of most plant organs (except for the aboveground parts of herbs) and MAP were significant (*P* < 0.01; Table 4).

**Table 4:** Regression relationships between caloric values (CAL, KJ g^–1^) of different plant organs and mean annual precipitation (MAP) of different ecosystem types.

	Functional groups	Organs	Regression equations	R^2^	*P*
Forests	Tree	Leaf	y = –0.0004x + 20.20	0.008	< 0.010
		Branch	y = –0.0006x + 19.99	0.029	< 0.001
		Trunk	y = –0.0004x + 19.49	0.017	< 0.001
		Root	y = –0.0014x + 20.56	0.180	< 0.001
		Leaf	–	–	0.924
	Shrub	Branch	y = –0.0005x + 19.66	0.044	0.001
		Root	y = –0.0018x + 18.13	0.007	< 0.001
		Leaf	–	–	0.152
	Herb	Stem	–	–	0.544
		Root	–	–	0.081
Grasslands	Herb	Aboveground	y = –0.0023x + 18.87	0.130	<0.010
		Underground	–	–	0.100
		Leaf	–	–	0.973
Deserts	Herb	Stem	–	–	0.966
		Root	–	–	0.607

## Discussion

### CAL significantly differs among different plant organs and vegetation types

This study confirmed that CAL significantly differs among different plant organs across various vegetation types, and that it is higher for aboveground organs (leaf, branch, and trunk) than for underground organs (root). Gao et al. [25] also reported that CAL in the leaf, branch, and other aboveground organs is higher than that in the root. Previous studies showed that the functional properties of plant organs are closely related to their nutrient contents [26]. The significant differences in the CAL of various plant organs recorded in this study verified that plants adopt different energy distribution and utilization strategies to adapt to changing environment and resource conditions. The aboveground parts of plants receive direct solar radiation for photosynthesis and contain large quantities of fat, starch, crude protein, and other high energy substances; thus, ensuring sufficient energy for the growth of other plant organs. In comparison, the roots are mainly responsible for absorbing minerals and moisture, rather than storing nutrients; consequently, the CAL of roots is often smaller than that of aboveground organs.

For forest vegetation, CAL significantly differed among various plant functional groups (trees > shrubs > herbs). Lin et al. [27] and Long [2] demonstrated that the CAL of plant organs increased with increasing light intensity. Adamandiadou et al. [28] showed that the CAL of leaves in the canopy of forests was higher than that of the leaves of shrubs and herbs. Forests have a distinct hierarchy; for instance, dominant tree species in forests have the optimal: position with respect to light competition, and receive more solar radiation. Shrubs are blocked by trees, and herbs are the lowest of all forests vegetation, receiving less solar radiation. Significant differences to plant CAL among different functional groups reflect the different strategies of how vegetation utilizes resources in different spatial niches.

Our study showed that the CAL of needle trees was significantly higher than that of broadleaved trees. Wang and Sun [29] showed that the CAL of needle trees in Xiaoxinganling is higher than that of broadleaved trees. Compared to subtropical and warm-temperate leaves, Tian et al. [30] reported that the leaf CAL of needle trees is generally higher than that of broadleaved trees. The differentiation of leaves into needles and broadleaves is the natural differentiation of trees in order to adapt to different environments. Needles improve the cold tolerance of trees. Other studies demonstrated that, compared to broadleaved trees, needle trees contain more resinous compounds and more high-heat substances, resulting in higher CAL [20, 31]. Therefore, the CAL of needle trees is generally greater than that of broad-leaved trees.

### Plant CAL does not significantly differ across regions

We found no significant variation in CAL of plant organs in different regions. A large number of studies have shown that environmental factors such as light intensity, soil type, moisture, and day length, directly and indirectly influence plant CAL [32–35]. Different ecological regions have different environmental conditions. To adapt to different light conditions, temperature, moisture, and other environmental conditions in different ecological regions, plant organs often adopt different energy strategies patterns. For instance, the distinctive anatomical differences between needle and broad-leaves allow them to respond to different environments. Furthermore, different plant organs have different energy distribution pattern. This significant difference in self-allocated energy might offset variation in the response of plants to environmental factors. Therefore, there is no significant variation in plant CAL in different regions. Of course, the lack of CAL data in some regions might result in uncertainty, requiring targeted research in the future.

### Plant CAL has inconsistent spatial pattern

There was no consistent longitude and latitude pattern for the CAL of different organs. For forests, the CAL of plant organs in the tree and shrub layer (except leaves) increased with increasing latitude, while the CAL in the roots of shrubs and herbs decreased with increasing longitude. The differences in latitudinal and longitudinal patterns among various organs may result from different environment adaptation strategies, or from the different control mechanisms of climate, such as MAT and MAP [36]. Our results showed that the CAL of most plant organs in forests had negative linear correlation with MAP and MAT, which might be explained by the fact that plants have to keep more energy in their organs to satisfy caloric demand and sustain self-growth in unsuitable environment with relative low temperature or insufficient precipitation [3, 19, 29]. However, there are limited basic data for the samples, such as lacking of soil properties and soil microorganisms data; thus, the possible mechanisms of the latitude and longitude pattern for the CAL of various organs remain unclear, which require to strengthen in the future.

### Potential for estimating biomass energy at different scales

CAL in different plant organs reflects the level of plant energy allocated to storage, and it is an important form of biomass energy [36]. Therefore, it is important to be able to document changes in CAL among different plant organs to estimate biomass energy at different scales. At present, some studies have explored plant CAL; however, most studies have focused on specific plant species and specific vegetation types [2, 3, 6, 8]. In this study, we detected large variation in CAL among different organs across different vegetation types. Thus, caution should be implemented when assuming consistent values across different organs, to estimate plant energy storage. Here, we developed a series of CAL reference values ​​for the different organs of plants that could be used to improve future estimates of biomass energy reserves. Such information is important for exploiting and utilizing biomass resources by policy-makers. Despite some limitations in the database, it was possible to capture slight fluctuations in plant CAL with respect to season [37], which should be emphasized in the future.

## Conclusions

This study established a comprehensive dataset of CAL for different plant organs in the terrestrial ecosystems of China. CAL significantly differed among plant organs (leaf, branch, trunk, and root) and vegetation types (forests, grasslands, and deserts). As expected, different strategies were detected in how energy was distributed across different organs. For example, the CAL of the aboveground organs is significantly larger than that of the underground parts, indicating that plants tend to store more energy in organs that produce energy. Furthermore, CAL noticeably varied among PFGs (trees, shrubs, and herbs) and plant life forms in forest vegetation. CAL was higher in needle trees compared to broadleaf tree, and was ordered: tree layer > shrub layer > herb layer. Therefore, due to the spatial niche differentiation in forests, plants have different strategies for using resources, and plants that receive sufficient light have higher CAL. However, there was no significant difference in the CAL of plant organs between different regions, indicating that the different patterns of energy distribution in the organs of different plants might offset the variation due to environmental differences. Overall, our findings demonstrate that CAL exhibits broad variation in different plant organs among different vegetation types, contrasting with the trends of carbon content in plant organs. Therefore, a set of CAL references ​​for different organs across different vegetation types is required to improve current estimates of biomass energy storage at regional and global scales.

## Supporting information

S1 Table Number of plant caloric values (CAL, KJ g^–1^) sampled in different ecosystem types. (PDF)

S1 Fig Frequency distribution of plant caloric values (CAL, KJ g^–1^) in forests. (PDF)

S2 Fig Frequency distribution of plant caloric values (CAL, KJ g–1) in deserts and grasslands. (PDF)

S3 Fig Changes to the caloric values (CAL, KJ g^–1^) of various plant organs in the different plant life forms (Needles vs. Broad-leaved). Different letters indicate significant differences in each cluster at the P = 0.05 level.
(PDF)

S4 Fig Changes to the caloric values (CAL, KJ g^–1^) among various plant organs in different plant life forms (Evergreen vs. Deciduous). Different letters indicate significant differences in each cluster at the P = 0.05 level.
(PDF)

S5 Fig Longitudinal variations of caloric values (CAL, KJ g^–1^) for different plant organs in forests. (PDF)

S6 Fig Longitudinal variations of caloric values (CAL, KJ g^–1^) of different plant organs in grasslands and deserts. (PDF)
