# Supplementary material for: Variation in the calorific values of different plants organs in China
Source: PLoS One. 2018 Jun 28;13(6):e0199762. doi: 10.1371/journal.pone.0199762 (PMC6023129; doi:10.1371/journal.pone.0199762)
Supplement: S1 Table — (PDF) [file pone.0199762.s001.pdf]

S1 Table

	Functional groups	Organs	Samples	Number of plant species
Forests	Tree	Leaf	949	930
		Branch	503	494
		Stem	554	552
		Root	281	273
	Shrub	Leaf	612	599
		Branch	254	252
		Root	151	150
	Herb	Leaf	390	381
		Stem	25	25
		Root	40	40
Grasslands	Herb	Aboveground	346	342
		Underground	90	88
Deserts	Herb	Leaf	61	61
		Stem	46	46
		Root	41	39
