# Supplementary figures and images for: Variation in the calorific values of different plants organs in China
Source: PLoS One. 2018 Jun 28;13(6):e0199762. doi: 10.1371/journal.pone.0199762 (PMC6023129; doi:10.1371/journal.pone.0199762)

S1 Fig.

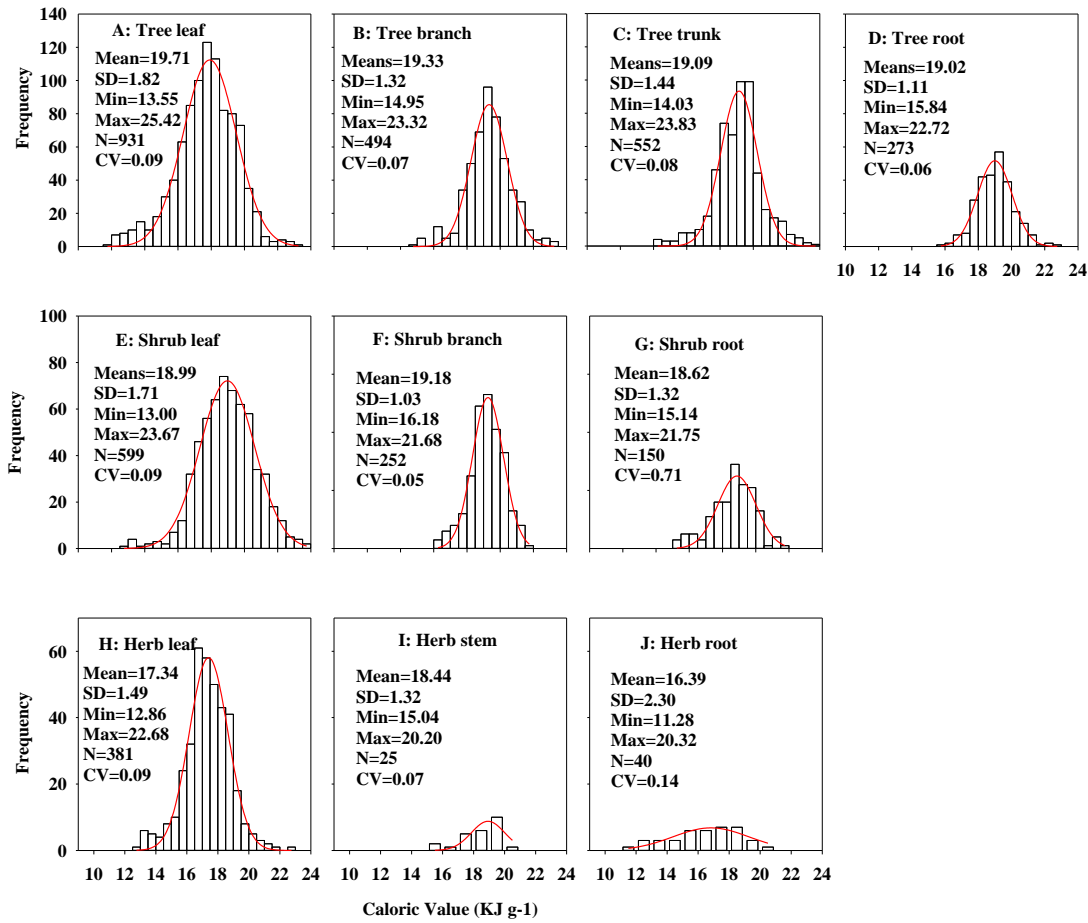

Supplement: S1 Fig — (PDF) [file pone.0199762.s002.pdf]

S2 Fig.

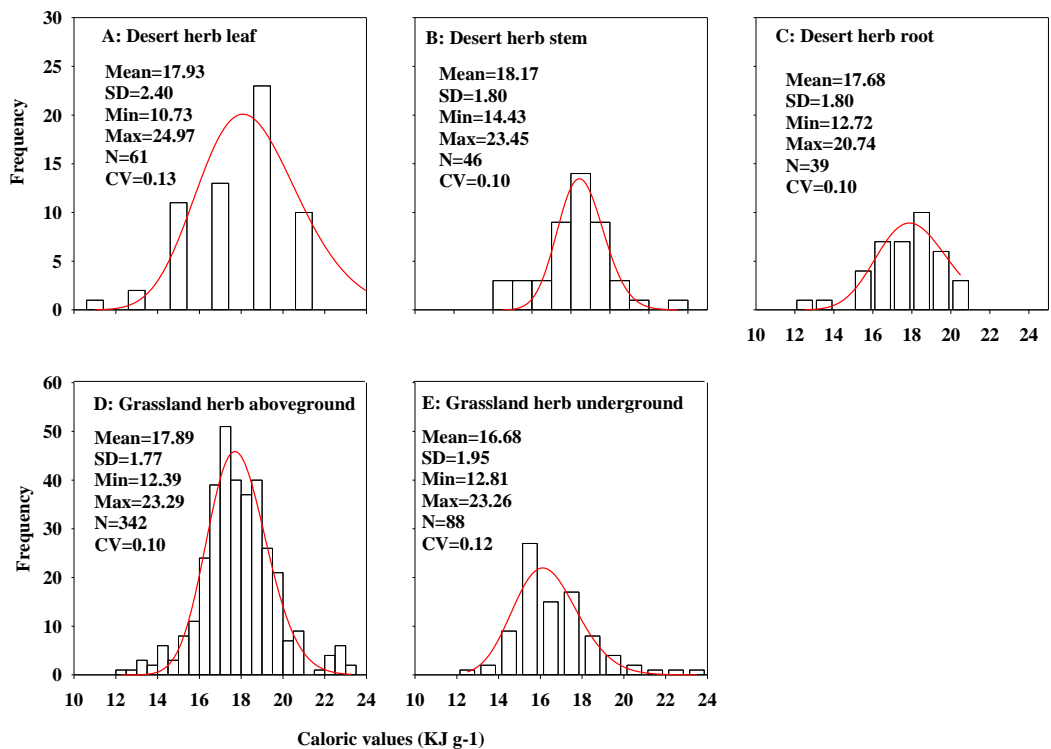

Supplement: S2 Fig — (PDF) [file pone.0199762.s003.pdf]

S3 Fig.

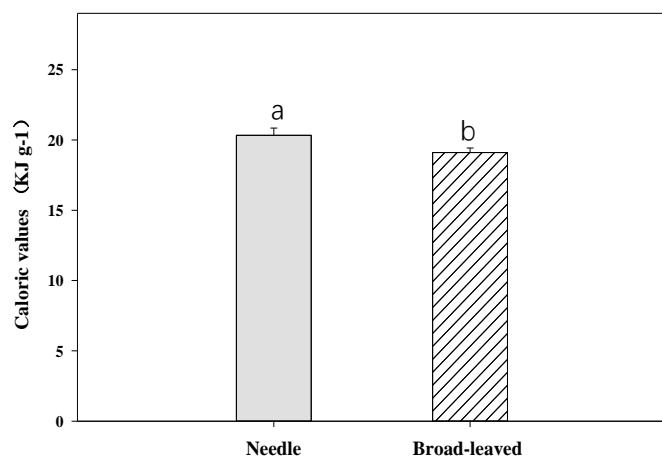

Supplement: S3 Fig — Different letters indicate significant differences in each cluster at the P = 0.05 level. (PDF) [file pone.0199762.s004.pdf]

S4 Fig.

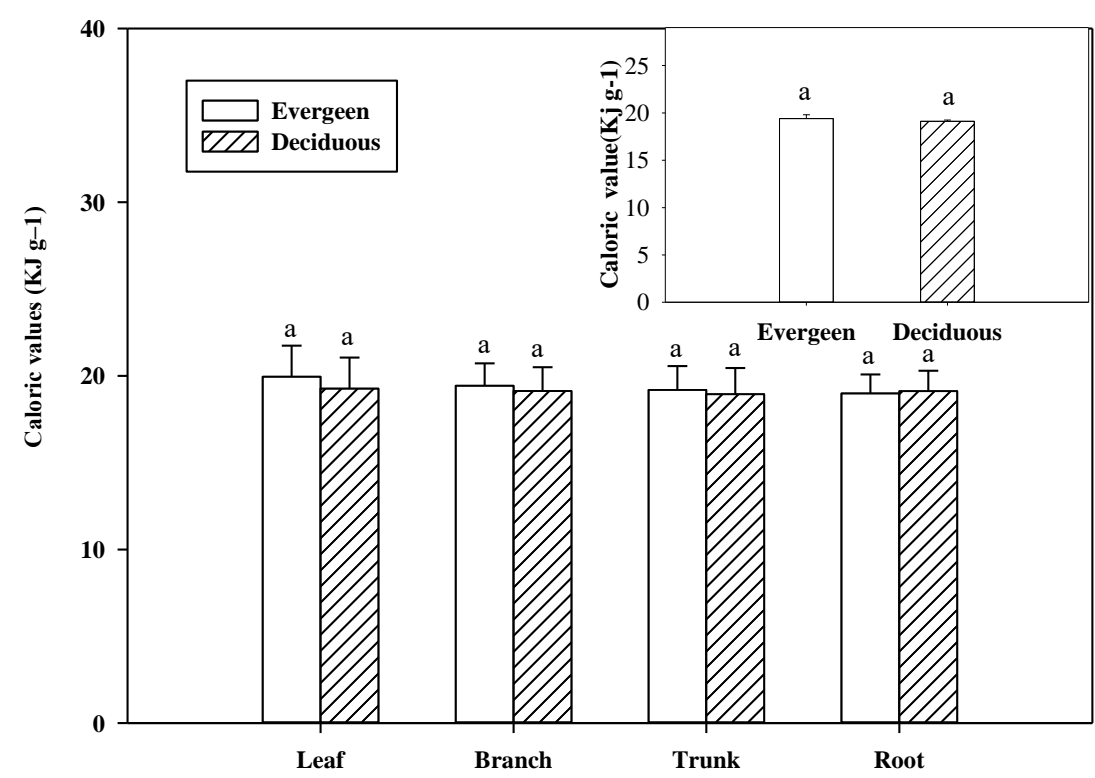

Supplement: S4 Fig — Different letters indicate significant differences in each cluster at the P = 0.05 level. (PDF) [file pone.0199762.s005.pdf]

S5 Fig.

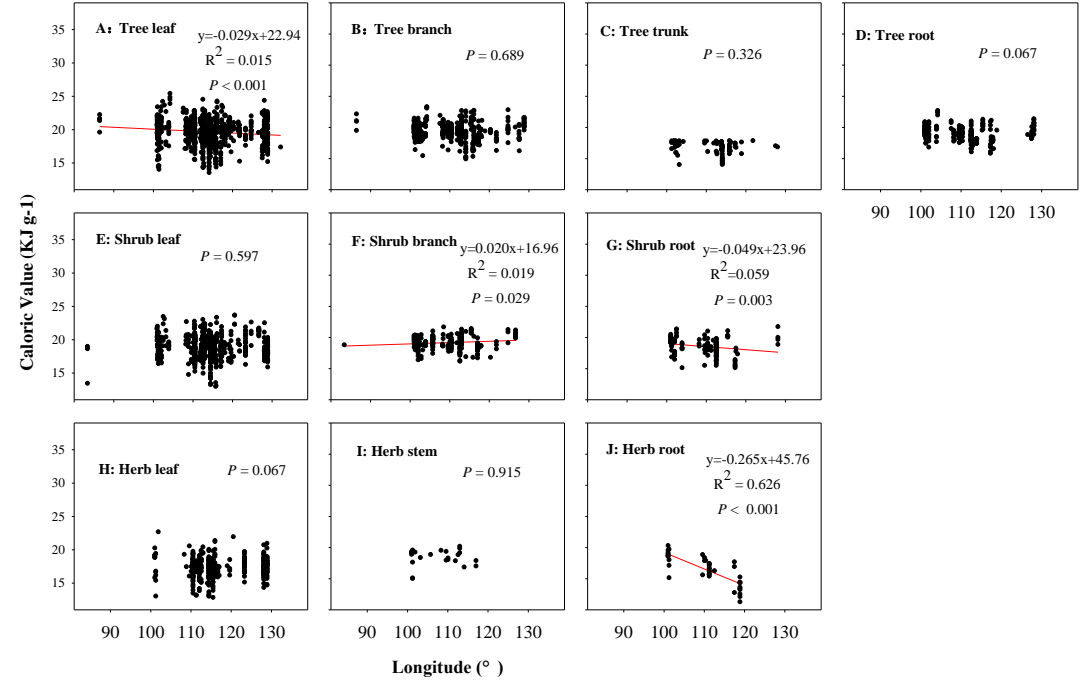

Supplement: S5 Fig — (PDF) [file pone.0199762.s006.pdf]

S6 Fig.

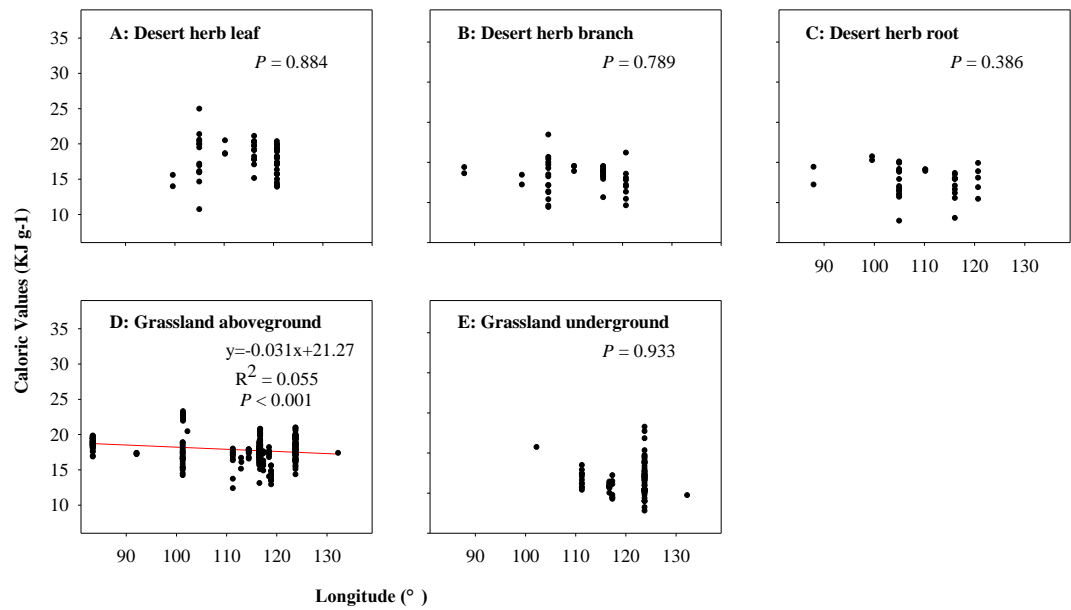

Supplement: S6 Fig — (PDF) [file pone.0199762.s007.pdf]
